# Human genetic polymorphisms in the Knops blood group are not associated with a protective advantage against *Plasmodium falciparum* malaria in Southern Ghana

**DOI:** 10.1186/1475-2875-12-400

**Published:** 2013-11-07

**Authors:** Helle H Hansson, Jørgen A Kurtzhals, Bamenla Q Goka, Onike P Rodriques, Francis N Nkrumah, Thor G Theander, Ib Christian Bygbjerg, Michael Alifrangis

**Affiliations:** 1 Centre for Medical Parasitology, Department of International Health, Immunology and Microbiology, Faculty of Health and Medical Sciences, University of Copenhagen, Copenhagen, Denmark; 2 Department of Clinical Microbiology, Copenhagen University Hospital (Rigshospitalet), Copenhagen, Denmark; 3 Department of Infectious Diseases, Copenhagen University Hospital (Rigshospitalet), Copenhagen, Denmark; 4 Department of Child Health, Korle Bu Teaching Hospital, PO Box KB 77, Korle Bu, Accra, Ghana; 5 Noguchi Memorial Institute for Medical Research, PO Box LG 581, Legon, Ghana

**Keywords:** Complement receptor 1, Plasmodium falciparum, Knops, Severe malaria

## Abstract

**Background:**

The complex interactions between the human host and the *Plasmodium falciparum* parasite and the factors influencing severity of disease are still not fully understood. Human single nucleotide polymorphisms SNPs associated with Knops blood group system; carried by complement receptor 1 may be associated with the pathology of *P. falciparum* malaria, and susceptibility to disease.

**Methods:**

The objective of this study was to determine the genotype and haplotype frequencies of the SNPs defining the Knops blood group antigens; Kn^a/b^, McCoy^a/b^, Swain-Langley1/2 and KCAM^+/-^ in Ghanaian patients with malaria and determine possible associations between these polymorphisms and the severity of the disease. Study participants were patients (n = 267) admitted to the emergency room at the Department of Child Health, Korle-Bu Teaching Hospital, Accra, Ghana during the malaria season from June to August in 1995, 1996 and 1997, classified as uncomplicated malaria (n = 89), severe anaemia (n = 57) and cerebral malaria (n = 121) and controls who did not have a detectable *Plasmodium* infection or were symptomless carriers of the parasite (n = 275). The frequencies were determined using a post-PCR ligation detection reaction-fluorescent microsphere assay, developed to detect the SNPs defining the antigens. Chi-square/Fisher’s exact test and logistic regression models were used to analyse the data.

**Results:**

As expected, high frequencies of the alleles Kn^a^, McC^b^, Sl2 and KCAM^-^ were found in the Ghanaian population. Apart from small significant differences between the groups at the Sl locus, no significant allelic or genotypic differences were found between the controls and the disease groups or between the disease groups. The polymorphisms define eight different haplotypes H1(2.4%), H2(9.4%), H3(59.8%), H4(0%), H5(25.2%), H6(0.33%), H7(2.8%) and H8(0%). Investigating these haplotypes, no significant differences between any of the groups were found.

**Conclusion:**

The results confirm earlier findings of high frequencies of certain CR1 alleles in Africa; and shed more light on earlier conflicting findings; the alleles McC^b^, Sl2, Kn^b^ and KCAM^-^ or combined haplotypes do not seem to confer any protective advantage against malaria infection or resulting disease severity. Based on these findings, in a very well-characterized population, malaria does not seem to be the selective force on these alleles.

## Background

In spite of the significant decrease in malaria-associated morbidity and mortality in recent years, approximately 216 million cases of malaria and an estimated 655.000 deaths were recorded in 2010 according to the World Malaria Report, 2011 [1]. Approximately 3.3 billion people were at risk of acquiring malaria in 2010 and it is, therefore, still considered one of the most common infectious diseases, particularly in sub-Saharan Africa where approximately four-fifths of all malaria cases worldwide are reported [1].

The complex interactions between the human host and *Plasmodium falciparum* and the factors influencing severity of disease are still not fully understood. Although never demonstrated *in vivo*, it is believed that the formation of rosettes; the spontaneous binding of *P. falciparum-*infected erythrocytes to uninfected erythrocytes [2], are associated with the pathology of *P. falciparum* malaria [3] through obstruction of the microcirculation [4-6]. Complement receptor 1 (CR1) is a polymorphic glycoprotein, expressed on a wide range of human cells, including erythrocytes [7]. The two major functions of the receptor are: 1) adherence to and removal of C3b- and C4b-containing immune complexes from the circulation, and 2) regulation of the complement cascade to prevent autologous complement attack [8]. The potential role of CR1 in the severity of malaria seems plausible since it has been shown that CR1 is involved in rosetting by binding to the *P. falciparum* erythrocyte membrane protein 1 (PfEMP1) [5].

Several polymorphic traits of CR1 have been reported; molecular weight polymorphisms, defined by deletion and duplication of sections of complement control proteins resulting in variations in the number of C3b binding sites [9]. In addition, an expression or density polymorphism has been found, defined by a single nucleotide polymorphism (SNP) in the intron of long homologous repeat D (LHR-D), determining the CR1 copy number expressed on the erythrocytes [10], however only in Caucasians, not in Africans [11,12]. Finally, polymorphisms have been found in the Knops blood group system carried by CR1, defined by SNPs located in the LHR-D [10,13,14]. Some SNPs in the Knops blood group system have been associated with rosetting [5,15] and possibly protection against malaria [16,17], and other SNPs with susceptibility to *P. falciparum* malaria [18].

Several antigens have been described in the Knops blood group system on CR1; Knops antigens a and b (Kn^a^/Kn^b^), rs41274768 [19], McCoy a and b (McC^a^/McC^b^), rs17047660 [20], Swain-Langley 1 and 2 (Sl1/Sl2), rs17047661 [20,21], KCAM + and - (KCAM^+^/KCAM^-^), rs6691117 [22], York + and - (Yka^+^/Yka^-^), rs3737002 [23] and Sl3,rs4844609 [21].

The Kn antigens are determined by a G (Kn^a^) or A (Kn^b^) at nucleotide position 4708, causing the amino acid (aa) change V1561M [19]. The McCoy antigens are determined by A (McC^a^) or G (McC^b^) at nucleotide position 4795 (K1590E change) [20]. Swain-Langley antigens are defined by an A (Sl1) or a G (Sl2) at nucleotide position 4828 (R1601G change) [20,21]. The KCAM antigens are defined by an A (KCAM^+^) or G (KCAM^-^) at position 4870 (I1615V change) [22].

The association between polymorphisms in the Knops blood group system and malaria has been investigated in several studies with focus on McC and Sl alleles. In 2000, Moulds *et al.* found that both the Sl2 and McC^b^ allele frequencies were very rare in European-Americans (n = 112, both Sl2 and McC^b^; 1%) compared to African-Americans (n = 150, 44% (Sl2) and 39% (McC^b^)), whereas the frequencies in a Malian population (n = 139) were 70% (Sl2) and 49% (McC^b^), and suggested that Sl2 might offer a protective advantage against severe malaria [16]. In 2001, Moulds *et al.* identified the molecular basis for the McC and Sl alleles and determined the frequencies in West Africa (samples grouped from Senegal, Sierra Leone, Ivory Coast and Ghana (total n = 182)) and another Malian population (n = 99). The allele frequencies were 79% (Sl2) and 31% (McC^b^) in pooled West African samples and 76% (Sl2) and 30% (McC^b^) in those from Mali [20]. The association between severe malaria and polymorphisms in the Knops blood group alleles McC and Sl was later studied in The Gambia [24,25] and in Kenya [17], but findings were contradictory. In The Gambia, neither the Sl2 nor the McC^b^ alleles were associated with protection against any form of severe malaria [24,25]. In the Kenyan study, the Sl2 allele and possibly the McC^b^ allele were associated with decreased susceptibility to cerebral malaria [17]. The KCAM and Kn alleles have not been studied as extensively. The prevalence of KCAM^+^ of 20% and Kn^b^ of <0.01% in African populations is taken from The Blood Group Antigen Facts Book [26].

A new SNP in the Knops blood group system was recently identified and 12 haplotypes were described, defined by this new SNP at position 4646A > G (Asn1540Ser) in combination with SNPs determining Kn, McC, Sl, Sl4, Sl5 and KCAM [27]. These haplotypes were subsequently studied by Fontes *et al*. in Brazil [18]. The H8 haplotype, defined by Kn^b^ and KCAM^-^, was found to be more frequent in individuals infected with *P. falciparum* (14%) compared to the controls (2%) [18]. Furthermore, the study found that the heterozygous Kn^a^/Kn^b^ genotype and the Kn^b^ allele were more frequent in patients with *P. falciparum* malaria compared to controls (28% *vs* 3.7% and 14% *vs* 2%, respectively) and the study indicated that the Kn^b^ allele and possibly the H8 haplotype are associated with susceptibility to *P. falciparum* infection, however, the sample size was very low; with only 25–35 individuals per group [18].

The purpose of the present study was to examine the possible association between the CR1 polymorphisms, Kn, McC, Sl and KCAM alleles, eight derived haplotypes and severity of malaria using samples from children between 0 and 15 years of age admitted to Korle-Bu Teaching Hospital in Accra, Ghana to confirm/disconfirm earlier studies.

## Methods

### Study population

The study population was children between 0 and 15 years of age admitted to the Department of Child Health, Korle-Bu Teaching Hospital, Accra, Ghana during the malaria season from June to August in 1995, 1996 and 1997. The general inclusion criteria were asexual *P. falciparum* parasitaemia of more than 10,000/μL and an axillary temperature of more than 37.5°C. Patients who fulfilled the criteria for uncomplicated malaria (UM, n = 89), severe malarial anaemia (SA, n = 57) or cerebral malaria (CM, n = 121), as described in detail elsewhere, were enrolled [28,29]. Patients with any other disease than malaria or with a positive sickling test were excluded as a criterion in the study, the samples were originally collected for, giving a total of n = 267 malaria patients. Control samples (CC, n = 275) were collected from healthy, sickle cell-negative children between 0 and 15 years of age with or without detectable *P. falciparum* in a thick blood smear from a nearby community, Dodowa. Both patients and controls were included after signed consent from patients or guardians after receiving standardized information in local language. In both Accra and Dodowa, the population is a mixture of several ethnic groups, possibly, with a slightly more uniform population in Dodowa. However, the dominating ethnic group in both Accra and Dodowa is Ga-Adangme. The study population is well described with very thorough patient characterization and has been studied extensively, among other things, with regards to interleukin 10, tumour necrosis factor and Mannose-binding lectin genotypes [28,29].The study was approved by the ethics and protocol review committee at the University of Ghana Medical School and Ministry of Health, Ghana.

### DNA extraction and whole genome amplification

Venous blood samples were collected in test tubes containing EDTA at admission. Plasma was separated within two hours after collection, and pellets were frozen at -20°C. Genomic DNA was extracted as described previously [30]. Whole genome amplification of the extracted products was performed with Repli-g Mini Kits (Qiagen, Copenhagen, Denmark).

### Polymerase chain reaction and sequencing

PCR primers were designed to amplify a region of *CR1* exon 29 based on NCBI Reference Sequence: NG_007481.1 which contains all four SNPs. Primers and conditions are provided in Table 1. Products were tested by agarose gel electrophoresis for size and specificity. One μl DNA was amplified in a 10 μl reaction consisting of 4.5 μl H_2_O, 2 μl AHPol HS 7.5 mM (AH Diagnostics AS, Aarhus, Denmark), and 2.5 μl 3 μM primermix. The reaction was performed in a 96-well PCR plate (Starlab GmbH, Hamburg, Germany) in a VWRi Duo Cycler (VWR/Bie&Berntsen, Radnor, PA, USA). Selected samples were sequenced to verify the genotyping of the Kna/b and KCAM^+/-^ alleles. Controls for the McC and Sl SNPs were kindly provided by Dr JoAnn Moulds.

**Table 1:** Primer sequences and conditions for the PCR and ligase detection reaction (LDR)

**Primers**	**Microsphere**	**Sequences**
		Fw 5’-TCTCTCAGTCATATCTTGTG-3’
**PCR**		Rw 5’-CTATTATGTTTATGCCAATTAA-3’
**Conditions**		**95°C 15 min, 40 cycles: 95°C 30 sec, 52°C 30 sec, 62°C 60 sec, 72°C 10 min**
**McC**^**a**^	039	5’-acaaatatctaactactatcacaaTCGGTGTATTTCTACTAAT**A**-3’
**McC**^**b**^	055	5’-acatcaaattctttcaatatcttcCGGTGTATTTCTACTAAT**G**-3’
**McC common**		5’-PHOS-AATGCACAGCTCCAGAAGTT-BIO-3’
**Sl1**	076	5’-tctcatctatcatactaattctttAGAAGTTGAAAATGCAATT**A**-3’
**Sl2**	095	5’-ttctttattctcattatcacatcaAGAAGTTGAAAATGCAATT**G**-3’
**Sl common**		5’-PHOS-GAGTACCAGGAAACAGGAGT-BIO-3’
**Kn**^**a**^	022	5’-caaacaaacattcaaatatcaatcAGAACAGCTGTTTGAGCTT**G**-3’
**Kn**^**b**^	037	5’-tacaacatctcattaacatatacaGAACAGCTGTTTGAGCTT**A**-3’
**Kn common**		5’-PHOS-TGGGAGAACGGTCAATATAT-BIO-3’
**KCAM**^**+**^	057	5’-acttacaataactactaatactctCTTTACCCTCACTGAGATC**A**-3’
**KCAM**^**-**^	078	5’-tttacaaatctaatcacactatacCTTTACCCTCACTGAGA**T**-3’
**KCAM common**		5’-PHOS-TCAGATTTAGATGTCAGCCC-BIO-3’
**LDR Conditions**		**95°C 1 min, 32 cycles: 95°C 15 sec, 60°C 2 min**

Primer sequences for the CR1 PCR and LDR conditions. The LDR (ligase-detection reaction) primers consist of set of three for each loci; two allele specific primers with anti-TAGs (small letters) matching the TAG sequence on the microsphere (Luminex xTAG microsphere number shown here), and one common primer marked with phosphate (PHOS) at the 5’ end and biotin (BIO) at the 3’ end. The site of the single nucleotide polymorphisms are highlighted and underscored.

### Genotyping

A genotyping assay was developed, based on the similar methods for the determination of cytochrome P450 2B6 alleles by Mehlotra *et al.*[31]; a post-PCR ligation detection reaction-fluorescent microsphere assay (LDR-FMA). Following the PCR, the products were used in a ligase detection reaction (LDR). One μl PCR product was added to 14 μl mastermix consisting of 0.15 μl 1 μM LDR primermix, 1.5 μl Ligase reaction buffer (New England Biolabs, Beverly, MA, USA: 20 mM Tris–HCl (pH 7.6), 25 mM potassium acetate, 10 mM magnesium acetate, 10 mM dithiothreitol, 1 mM NAD+, 0.1% Triton X-100), 0.05 μl (2 units) Taq DNA ligase (New England Biolabs, Beverly, MA, USA) and 12.3 μl H_2_O pr. sample. The LDR primermix consisted of three primers pr SNP; two allele-specific primers and one common. The common primer was 5’-phosphorylated and 3’-biotinylated and unique 24 bp tag sequences were attached to each allele-specific primer at the 5’ end by the manufacturer (MWG Biotech Ebersberg, Germany). All the LDR primers were designed based on NCBI Reference Sequence: NG_007481.1 and the tags were chosen as complementary to anti-tag sequences attached to unique fluorescent Luminex MagPlex-TAG™ microspheres (Luminex Corporation, Austin, TX, USA). A biotin-labelled product will only be formed by the allele-specific and common primer due to the 3’ mismatched base. Tag sequences, LDR primers and conditions are shown in Table 1. The reaction was performed in a 96-well PCR plate (Starlab GmbH, Hamburg, Germany) in a VWRi Duo Cycler (VWR/Bie&Berntsen, Radnor, PA, USA). In a following hybridization reaction 5 μl LDR product was incubated with 68 μl TMAC solution (3 M tetramethyl-ammonium chloride, 50 mM Tris–HCl (pH 8.0), 3 mM EDTA (pH 8.0), 0.1% SDS) containing 800 of each microsphere, for 5 min at 96° in a 96-well PCR plate (Starlab GmbH, Hamburg, Germany) in a VWRi Duo Cycler (VWR/Bie&Berntsen, Radnor, PA, USA) and 40 min at 37°C shaking in darkroom 500 rpm. Six μl TMAC solution containing 120 0.12 μg/ml streptavidin-R-phycoerythrin conjugate (Sigma Aldrich, St. Louis, MO, USA) was added to each well and incubated at 37°C shaking in darkroom 500 rpm for 40 min. The plate was analysed at 37°C on the Bio-Plex suspension array system using Luminex xMAP technology and Bio-Plex Manager analytical software ver. 6.1 (Bio-Rad Laboratories, Hercules, CA, USA). The microspheres were sorted according to their unique fluorescence and the phycoerythrin signal is measured as median fluorescence intensity (MFI); 50 of each microsphere were analysed.

### Statistical analyses

Deviations from the Hardy-Weinberg equilibrium were tested using the Court Lab Calculator [32]. Cut-off was set to p < 0.05. Any locus with deviations from the Hardy-Weinberg equilibrium would be interpreted with caution in the further analysis. Chi-square or Fisher’s exact test (SigmaPlot 12.3 SPSS Inc., USA) was used to compare allele and genotype frequencies of the different groups. Logistic regression models were used to determine associations between malaria disease severity and prevalence of Knops blood group polymorphisms. Several analysis were done to determine odds ratios and p values, with outcome variables defined as disease outcome (defined as the four groups; CC, UM, SA and CM) and parasitaemia (as a continuous variable and categorical variable (parasitaemia > 200,000/uL – yes/no). Age and gender were included as covariates. P-values ≤0.05 were considered significant.

Calculations were performed using SAS ver. 9.2, (2002–2008 SAS Institute Inc, Cary, NC, USA). Haplotypes were determined based on the definitions described by Covas *et al.*[27]. Chi-square test or Fisher’s exact test was used to determine differences between the control group and the CC, SA and CM groups and between the distributions in Ghana and Brazil. Individuals heterozygous at more than one locus (15%, n = 82) were excluded from this part of the analysis and analysed using SigmaPlot 12.3 SPSS Inc, USA.

## Results

### Population demographics

Samples were collected from children from 0 to 15 years of age. No significant differences were observed in the mean age or the proportion of males between the control and disease groups except for the severe anaemia group where the mean age was lower than controls (P = 0.93 ) and the proportion of males was higher than controls (P = 0.02) (Table 2).

**Table 2:** Demographic data for the Ghanaian study population

	**Controls (n = 275)**	**Uncomplicated malaria (n = 89)**	**Severe anaemia (n = 57)**	**Cerebral malaria (n = 121)**
**Age (years)**				
**Mean ± SD**	8.02 ± 3.99	6.00 ± 3.40	3 .00 ± 2.43	5.00 ± 2.87
**Minimum/maximum**	0-15	1-14	0-9	1-13
**Sex (n)**				
**Male**	138 (50.2 %)	48 (54.0 %)	39 (68.4 %)	66 (54.5 %)
**Female**	137 (49.8 %)	41 (46.0 %)	18 (31.6 %)	55 (45.5 %)
**Parasitaemia (p/ul)**				
**Median**	-	49.700	51.224	97.614
**Percentiles 25 and 75**	-	24.360-116.156	17.760-117.806	34.010-212.800
**Haemoglobin (g/dl)**				
**Mean ± SD**	-	10.4 6 ± 1.93	4.00 ± 0.73	7.54 ± 2.17
**Minimum/maximum**	-	6.80-17.50	1.80-5.00	1.90-13.40

Demographic data for the Ghanaian control group and disease groups; uncomplicated malaria, severe anaemia and cerebral malaria. The significance level was set to p < 0.05, when comparing the groups to the control group.

### Allele and genotype frequencies at the Kn McC, Sl and KCAM loci in the disease and control groups

The capacity of the Luminex technique to correctly detect the SNPs at the Kn, McC, Sl and KCAM loci was tested by use of control DNA samples from individuals with known genotypes. The assay correctly identified the various SNPs in controls and the MFI of positive readouts were easily distinguished from the background reading obtained in control negative samples. Results were confirmed with sequencing of selected samples.

The allele distribution was determined at the four loci: Kn, McC, Sl and KCAM. All were in Hardy-Weinberg equilibrium, except for the Sl locus (Χ^2^ = 18.61, p < 0.0001), due to a higher frequency of Sl1/lower frequency of Sl1/Sl2 than expected. Analysing Hardy-Weinberg in the different groups at the Sl locus, showed that the CC group (Χ^2^ = 4.12, p = 0.04), the SA group (Χ^2^ = 4.86, p = 0.03) and the CM group (Χ^2^ = 9.57, p = 0.002) all deviated from Hardy Weinberg, whereas the UM group (Χ^2^ = 1.62, p = 0.20) was the only one in Hardy-Weinberg equilibrium. The deviations in three of the groups were very small and seemed to reflect a small anomaly, so the Sl locus was not excluded from further analysis. The distribution in the UM, SA and CM groups was compared to the CC group and to each other. At the Sl locus, the UM group differed significantly from the CC and SA groups and showed a tendency to differ from the CM group with a higher frequency of the Sl2 allele (UM *vs*: CC p = 0.01, SA p = 0.03 and CM p = 0.08) (Table 3). Otherwise, no significant differences were seen at the other loci when comparing all groups; Kn (p > 0.5), McC (p > 0.7), KCAM (p > 0.1) and when comparing groups two by two.

**Table 3:** Prevalence of CR1 alleles and genotypes at four loci; Kn, McC, Sl and KCAM

**Frequency of the genotypes n (%)**		**Controls (n = 275)**	**Uncomplicated malaria (n = 89)**	**Severe anaemia (n = 57)**	**Cerebral malaria (n = 121)**
**Kn**^**a/b**^	**a/a**	275 (100)	88 (98.9)	57 (100)	120 (99.2)
SNP Rs41274768	**a/b**	0 (0)	1 (1.1)	0 (0)	1 (0.8)
	**b/b**	0 (0)	0 (0)	0 (0)	0 (0)
**Allele frequency Kn**^**b**^		0.0	0.006	0.0	0.004
**McC**^**a/b**^	**a/a**	147 (53.5)	52 (58.4 %)	30 (52.6)	68 (56.2)
SNP Rs17047660	**a/b**	109 (39.6)	26 (29.2)	21 (36.8)	42 (34.7)
	**b/b**	19 (6.9)	11 (12.4)	6 (10.5)	11 (9.1)
**Allele frequency McC**^**b**^		0.27	0.27	0.29	0.27
**Sl1/Sl2**	**1/1**	15 (5.5)	2 (2.2)	5 (8.8)	8 (6.6)
SNP Rs17047661	**1/2**	74 (26.9)	14 (15.7)	13 (22.8)	24 (19.8)
	**2/2**	186 (67.6)	73 (82.0)	39 (68.4)	89 (73.6)
**Allele frequency Sl2**		0.81	0.90	0.80	0.83
**KCAM**^**+/-**^	**+/+**	5 (1.8)	0 (0)	0 (0)	0 (0)
SNP Rs6691117	**+/-**	45 (16.4)	10 (11.2)	8 (14.0)	24 (19.8)
	**-/-**	225 (81.8)	79 (88.8)	49 (86.0)	97 (80.2)
**Allele frequency KCAM**^**-**^		0.90	0.94	0.93	0.90

Prevalence of the alleles and genotypes at the four loci, Kn, McC, Sl and KCAM, determined in a Ghanaian population, divided in four groups; controls (CC), uncomplicated malaria (UM), severe anaemia (SA) and cerebral malaria (CM).

a/b, 1/2 and +/– denotes the alleles at the four loci.

The genotype frequencies at the four loci in the different groups are shown in Table 3. In the study group, the Kn^b^ allele was very rare and only identified in two Kn^a/b^ heterozygotes (0.4%). The McC^b^ allele was more common with a total frequency of 8.7% and 35.5% in the McC^b/b^ and the McC^a/b^ genotype, respectively. The Sl2 and KCAM^-^ alleles were very common with high frequencies of both the homozygotes (Sl2/2 71.4%, KCAM^-/-^ 83.0%) and the heterozygotes (Sl1/2 23.1%, KCAM^+/-^ 16.1%). Logistic regression, adjusted for age and sex, tested for an association between the genotypes and parasitaemia or disease outcome (defined as UM, SA and CM). No significant associations were found between any genotype at the four loci and level of parasitaemia (p > 0.1) and between any genotype and disease outcome (p > 0.3).

Furthermore, the allele frequencies for McC and Sl were compared against earlier findings from studies in various African countries (Table 4). The Ghanaian study population differed significantly from the Gambian at the McC loci by having a lower frequency of the McC^b^ allele (p < 0.001, 0.27 *vs* 0.39). At the Sl loci Ghana differed from both Mali (p = 0.02, 0.83 *vs* 0.76) and Kenya (p < 0.001, 0.83 *vs* 0.68) by a higher frequency of the Sl2 allele and slightly differed from The Gambia (p = 0.06, 0.83 *vs* 0.80). Only few data for the KCAM and Kn loci are known. At the KCAM loci, Ghana showed a higher frequency of the KCAM^-^ allele compared to an earlier publication (p = 0.045, 0.91 *vs* 0.80). No differences were found at the Kn loci.

**Table 4:** **McC and S**l **alleles in various Africa populations**

**Population**	**McC**^**a**^**/McC**^**b**^	**Sl1/Sl2**
**Ghana (n = 542)**	0.73/0.27	0.17/0.83
**Kenya**^**1 **^**(n = 460)**	0.71/0.29	0.32/0.68**
**The Gambia**^**2 **^**(n = 853)**	0.61/0.39**	0.20/0.80
**Mali**^**3 **^**(n = 99)**	0.69/0.31	0.24/0.76*
**West Africa**^**3 **^**(n = 182)**	0.69/0.31	0.21/0.79

Allele frequencies found in African populations for the Knops blood group alleles at the loci; McC and Sl. All studies were compared to the frequencies found in Ghana. * and ** differed significantly from the distribution seen in Ghana (p < 0.02) and (p < 0.001), respectively. ^1^Thathy *et al.*[17], ^2^Zimmerman *et al.*[24] , ^3^Moulds *et al.*[20]. The frequencies shown reflect pooled cases and controls.

a and b denotes the alleles at the McCoy (McC) locus, 1 and 2 denotes the alleles at the Swain-Langley (Sl) locus.

### Distribution of CR1 haplotypes in the disease and control groups

The SNPs of the four loci were constructed into haplotypes. The frequencies and definitions of the eight haplotypes are shown in Table 5. The most prevalent haplotypes were the H3 (60%), H5 (25%) and H2 (9%). H1, H6 and H7 ranged from (0.3-3%), and H4 and H8 were not found.

**Table 5:** Haplotype frequencies of the four SNPs in the CR1 gene, determined in Ghanaian and Brazilian study populations

**Disease group (alleles)**	**Haplotype definitions**							
	**H1 Kn**^**a **^**McC**^**a **^**Sl1 KCAM**^**+**^	**H2 Kn**^**a **^**McC**^**a **^**Sl1 KCAM**^**-**^	**H3 Kn**^**a **^**McC**^**a **^**Sl2 KCAM**^**-**^	**H4 Kn**^**a **^**McC**^**b **^**Sl1 KCAM**^**-**^	**H5 Kn**^**a **^**McC**^**b **^**Sl2 KCAM**^**-**^	**H6 Kn**^**a **^**McC**^**b **^**Sl2 KCAM**^**+**^	**H7 Kn**^**a **^**McC**^**a **^**Sl2 KCAM**^**+**^	**H8 Kn**^**b **^**McC**^**a **^**Sl1 KCAM**^**-**^
**Controls (n = 448)**	0.02	0.10	0.59	0.00	0.24	0.01	0.03	0.00
**Uncomplicated malaria (n = 163)**	0.01	0.07	0.63	0.00	0.28	0.00	0.02	0.00
**Severe anaemia (n = 98)**	0.04	0.11	0.56	0.00	0.28	0.00	0.01	0.00
**Cerebral malaria (n = 205)**	0.03	0.08	0.60	0.00	0.25	0.00	0.03	0.00
**Ghana (n = 914)**	0.02	0.09	0.60	0.00	0.25	0.003	0.03	0.00
**African Brazilians**^**1 **^**(n = 86)**	0.14	0.15	0.42	0.12	0.07	0.070	0.023	0.11
**European Brazilians**^**1 **^**(n = 66)**	0.73	0.15	0.015	0.00	0.015	0.00	0.00	0.015
**Asian Brazilians**^**1 **^**(n = 84)**	0.79	0.21	0.00	0.00	0.00	0.00	0.00	0.00

^1^Covas *et al.*[27] SNPs at the four loci Kn, McC, Sl and KCAM can define eight different haplotypes. There was no difference between the disease groups in the Ghanaian population with H3 and H5 having the highest frequencies, while H4 and H8 were not found. The Ghanaian groups were also compared to the three Brazilian populations. All differed significantly from Ghana p < 0.001. When constructing the haplotypes, individuals heterozygote at more than one locus was excluded from the analysis (15 %, n = 82).

a/b, 1/2 and +/– denotes the alleles at the four loci.

When comparing the distributions of the haplotypes in the four groups, no significant differences were found between any of the groups (p > 0.3). A study in three Brazilian groups: European Brazilian, Asian Brazilians and African Brazilians defined the CR1 haplotypes [27]. All three groups differed significantly from the total Ghanaian study group (p < 0.001) and the Ghanaian control group alone (p < 0.001). The distribution in the African Brazilians differed from the Ghanaians by higher frequencies of H1, H2, H4, H6, H7 and H8. H4 and H8 were not found in Ghana at all. H3 had the highest frequency in Brazilians (42%) as in Ghana (60%), whereas the distribution of the other haplotypes was very different (Table 5). A total of 82 individuals (15%) were not included in the haplotype analysis since they were heterozygote at more than one locus.

## Discussion

Based on the described pathogenetic and epidemiological evidence, it was hypothesized that there would be an association between polymorphisms in the Knops blood group system and malaria severity. In this present study, no evidence was found for such an association.

The McC and Sl-polymorphisms have previously been studied in African populations including Mali [16,20], a pool of West African countries (including Senegal, Guinea, Sierra Leone, Ivory Coast and Ghana [20]), Kenya [17] and The Gambia [24]. Similar to these studies, these current findings showed higher frequencies of the McC^b^ (27% *vs* 1%) and Sl2 (83% *vs* 1%) alleles compared to Caucasian populations [16]. Kn and KCAM have not been studied as extensively, however, this study showed an elevated KCAM^-^ frequency compared to Caucasians (91% *vs* 2%), while frequencies of the Kn^a^ were similar in Africans and Caucasians concurring with an earlier publication [26]. The UM group differed significantly from the CC and SA group and borderline significantly from the CM group by having a higher frequency of the Sl2 allele. This unexplainable difference might be caused be the small anomaly causing the deviation from the Hardy-Weinberg equilibrium (where only the UM group was in equilibrium). Thus, any conclusions based on this should be made with caution. Furthermore, no associations were found between the genotypes and the severity of malaria.

From the various alleles at the four loci, eight different haplotypes can be constructed based on definitions described in Covas *et al.*[27]. Six of the eight haplotypes were found in the Ghanaian population while in another study describing these haplotypes, eight haplotypes were found in an African-Brazilian population [27]. H2 and H3 were among the most prevalent haplotypes in Ghana; while the prevalence of H5 was much lower here than in Brazil. The distribution of the haplotypes in Ghana was significantly different from the African-Brazilian population, but that can most likely be explained by the differences between populations. In the present study, no significant association between haplotypes and malaria disease outcome was found. A number of individuals were excluded from the haplotype analysis since they were heterozygote at more than one locus. Even though this is a high percentage of the total number of individuals, the remaining sample size are believed to be sufficient to provide a true reflection of the haplotype distribution in the Ghanaian population.

Thus, it seems that the polymorphisms are not associated with any of the studied complications of falciparum malaria. Earlier studies have shown conflicting data when investigating the possible association between the McC and Sl polymorphisms and malaria [17,24]. Several factors could have caused the contradictory findings between studies; ethnic differences in the study populations, differences in study design or area differences, e g, differences in malaria transmission intensity, however, the two largest studies; this current study (n = 542) and the study in The Gambia (n = 853) [24] found no association between the CR1 polymorphisms and malaria. Also variations in CR1 expression levels could be taken into consideration. Recently, Thomas *et al.* showed that low CR1 copy numbers on the erythrocytes could affect the ability to remove circulating immune complexes, one of the most important functions of CR1 [8], in children from Mali with severe malaria [33].

Conversely, malaria may not be the driving force behind the selective pressure of CR1 polymorphisms. Recently, Tetteh-Quarcoo *et al.* tested different recombinant constructs of CR1 and showed that the selective pressure on the Sl2 and McC^b^ alleles does not appear to be *P. falciparum* malaria since no differences in complement-regulatory functionality, rosetting, or binding to PfEMP1 were found between the alleles [34]. Based on these recent findings, the study from The Gambia [24], and this current study, the polymorphisms in the CR1 Knops blood group system appear not to be associated with the severity of malaria. Furthermore, Noumsi *et al*. showed that the alleles McC^b^ and Sl2 are associated with resistance to *Mycobacterium tuberculosis* infection in a Malian population [35], which supports the theory of a selective pressure on the CR1 alleles, unrelated to malaria.

## Conclusion

The results from this study confirm earlier findings of high frequencies of certain CR1 alleles in Africa and shed more light on the conflicting earlier findings: McC^b^ and Sl2, and Kn^b^ and KCAM^-^ do not seem to confer any protective advantage against malaria infection or resulting disease severity. Furthermore, combining the loci into haplotypes showed no significant association with disease severity either. Based on these findings, in a very well-characterized population, malaria does not seem to be the selective force on these alleles.

## Abbreviations

CC: Controls; CM: Cerebral malaria; CR1: Complement receptor one; FMA: Fluorescent microsphere assay; Kn: Knops antigen; LDR: Ligase detection reaction; LHR-D: Long homologous repeat D; McC: McCoy; MFI: Median fluorescent intensity; PfEMP1: *Plasmodium falciparum* erythrocyte membrane protein one; PCR: Polymerase chain reaction; SA: Severe anaemia; Sl: Swain-Langley; SNP: Single nucleotide polymorphism; TMAC: Tetramethyl-ammonium chloride; UM: Uncomplicated malaria.

## Competing interests

The authors declare that they have no competing interests.

## Authors’ contributions

HHH acquired and analysed the data, carried out the laboratory work and wrote the manuscript. MA, JAK and ICB contributed to study design and interpretation of data, supervised DNA work and revised the manuscript for important intellectual content. QBG, OPR, FNN and TGT contributed to concept development and study design, edited and revised the manuscript critically for content. All authors read and approved the final manuscript.
